# Comparative analysis of diagnostic and predictive performance of novel renal biomarkers in plasma and urine of acute kidney injury patients

**DOI:** 10.1186/2197-425X-3-S1-A258

**Published:** 2015-10-01

**Authors:** G Schley, C Köberle, E Manuilova, S Rutz, R Kientsch-Engel, K-U Eckardt, C Willam

**Affiliations:** University Hospital Erlangen, Erlangen, Germany; Roche Diagnostics GmbH, Penzberg, Germany

## Intr

Renal biomarkers represent an attractive new diagnostic tool which not only implies early diagnosis of AKI, but provides also information about patients at risk for AKI and prediction of adverse clinical outcome parameters like the need for renal replacement therapy, length of stay in ICU and mortality. Biomarker levels in urine however may be altered by intravascular volume availability and chronic renal insufficiency.

## Objectives

This study directly compared the diagnostic and prognostic performance of biomarkers in urine and plasma.

## Methods

This prospective cohort study included 110 unselected adults undergoing cardiac surgery. Plasma and/or urine concentrations of creatinine, cystatin C, neutrophil gelatinase-associated lipocalin (NGAL), liver fatty acid-binding protein (L-FABP), kidney injury molecule 1 (KIM1), and albumin and furthermore a multiplex panel of 15 biomarkers in plasma and urine were measured during the perioperative period. The primary outcome was AKI defined by AKIN criteria.

## Results

Biomarkers in plasma showed markedly better predictive and diagnostic performance than their urinary counterparts. Discriminative power of urinary biomarkers improved when concentrations were related to urinary creatinine but still did not achieve AUC values of markers measured in plasma samples. Before surgery plasma IP10 (interferon-γ-induced protein 10, CXCL 10), cystatin C and MIG (monokine induced by interferon-γ, CXCL9) best predicted postoperative AKI (AUC 0.73-0.70). Best diagnostic performance 4h after surgery had NGAL (AUC 0.83), cystatin C (0.76), and MIG (0.74) in plasma. Combination with clinical scores (EuroSCORE and Cleveland Clinic Foundation Score) or combinations of several biomarkers did not significantly improve predictive or diagnostic power of either plasma or urine markers.

## Conclusions

In our cohort plasma biomarkers had higher discriminative power to predict and to diagnose AKI than urine biomarkers. Plasma IP10 and NGAL performed best in predicting and diagnosing AKI respectively. Their performance could not be improved by combining with clinical scores or additional biomarkers.

